# Revolutionizing Autoimmune Research: The Role of Caveolin‐1

**DOI:** 10.1002/iid3.70230

**Published:** 2025-08-08

**Authors:** Yanan Gao, Liangyu Mi, Yingren Deng, Zhaojun Jia, Miaomiao Zhao, Yuting Hu, Yuli Ji, Xiaoyao He, Ke Xu

**Affiliations:** ^1^ Third Hospital of Shanxi Medical University, Shanxi Bethune Hospital, Shanxi Academy of Medical Sciences, Tongji Shanxi Hospital Taiyuan China; ^2^ Jiexiu People's Hospital Jinzhong China

**Keywords:** autoimmune disease, biomarker, caveolin‐1, immunity, inflammation

## Abstract

**Introduction:**

Caveolins (Cav) include Cav‐1, Cav‐2, and Cav‐3, with Cav‐1 being the most studied due to its prominent role as a major component of plasma membrane caveolae. Cav‐1 is involved in a wide range of cellular functions and plays a key role in regulating signaling pathways related to immune responses and inflammation. Recently, research on Cav‐1 in autoimmune diseases (AIDs) has garnered significant interest.

**Methods:**

This paper provides an overview of the research on Cav‐1's involvement in AIDs, including rheumatoid arthritis, systemic lupus erythematosus, Sjögren syndrome, anti‐neutrophil cytoplasmic antibody‐associated vasculitis, systemic sclerosis, connective tissue disease‐associated interstitial lung disease, autoimmune disorders of the nervous system, autoimmune uveitis, autoimmune thyroid disease, and autoimmune myocarditis.

**Results:**

Cav‐1 plays a critical role in various AIDs, acting as a key protein in inflammatory and immune cells. It regulates multiple signaling processes by controlling the translocation of signaling molecules and modulating various pathways. Cav‐1 is increasingly recognized as a biomarker in certain AIDs and may become pivotal in treating these diseases in the future.

**Conclusion:**

Cav‐1 is a crucial player in the pathogenesis of many AIDs and has the potential to serve as both a diagnostic marker and a therapeutic target for these diseases. As research into Cav‐1 deepens, it may offer new insights into the diagnosis, treatment, and drug sensitivity of AIDs, emerging as a promising target for future therapeutic strategies.

## 1 Introduction

Caveolae, also referred to as cytoplasmic crypts, are small (50–100 nm) invaginations of the plasma membrane, first identified by Palade. Their name, derived from the Latin word for “small caves,” comes from their characteristic appearance under a transmission electron microscope [1]. These structures are specialized lipid rafts, rich in sphingolipids and cholesterol, and they are crucial for the proper functioning of cells. The primary protein associated with caveolae is Caveolin (Cav), which is considered the most critical component of these invaginations [2]. Cav, often described as the “shell protein” of the caveolae, is predominantly located at the plasma membrane and is widely expressed in mammals, although the levels of expression vary across different tissues. In addition to Cav, caveolae are associated with a family of proteins known as Cavins. These proteins contribute to the regulation of caveolae by assisting in membrane remodeling and aiding in the transport of Cav and other associated structures [3]. Cav is instrumental in a wide range of biological processes, including immune responses, endocytosis, cholesterol homeostasis, signal transduction, and tumor suppression [4]. It is crucial for the formation of caveolae, mediates endocytosis, helps maintain cellular cholesterol balance, and facilitates interactions with a range of signaling molecules through its Caveolin scaffolding domain (CSD). These interactions are vital for regulating cellular signaling pathways that influence cell proliferation, apoptosis, transformation, and carcinogenesis [5]. A deficiency in Cav can lead to the development of various diseases, such as cancer, lung disorders, neurodegeneration, muscular dystrophy, cardiomyopathy, and atherosclerosis. Given its pivotal role in these conditions, serum Cav becomes a promising biomarker for forecasting disease progression and outcomes. For instance, increased serum Cav‐1 levels have been recognized as a potential prognostic marker for both prostate cancer and melanoma [6, 7]. Additionally, Cav‐1 levels can provide insights into the progression of colorectal cancer [8], while Cav‐3 levels are associated with cardiac hypertrophy, systolic dysfunction, new‐onset heart failure, and arrhythmias [9, 10].

Autoimmune diseases (AIDs) are complex and chronic disorders that arise from the interplay of genetic, environmental, and immune system factors. It is characterized by an abnormal immune response where the body's immune system attacks its own tissues and organs. The worldwide prevalence of AIDs is estimated to fall between 7.6% and 9.4% [11], with significant variation across different ages, sexes, and geographic regions. These diseases can affect various organs or be systemic in nature, contributing substantially to both morbidity and mortality rates [12]. Standard treatments for AIDs include glucocorticoids (GCs) and conventional disease‐modifying antirheumatic drugs. However, these therapies are often associated with prolonged treatment periods and a range of side effects. In recent years, advancements in the understanding of AIDs pathogenesis have paved the way for more targeted therapeutic approaches, including biologics and small‐molecule inhibitors of immune signaling pathways. Cav is a central protein that is essential in modulating immune signaling pathways and inflammatory responses during the development of AIDs [13]. These pathways are crucial for the activation and differentiation of immune cells, which directly influence the autoimmune response. Moreover, a deficiency in Cav‐1 is linked to increased infiltration of inflammatory cells and enhanced production of inflammatory cytokines [14]. Cav also plays a significant role in the proliferation and migration of immune cells. Consequently, targeting Cav in therapeutic strategies may offer a promising new treatment option for AIDs.

## 2 Cav Family

**Figure 1:**
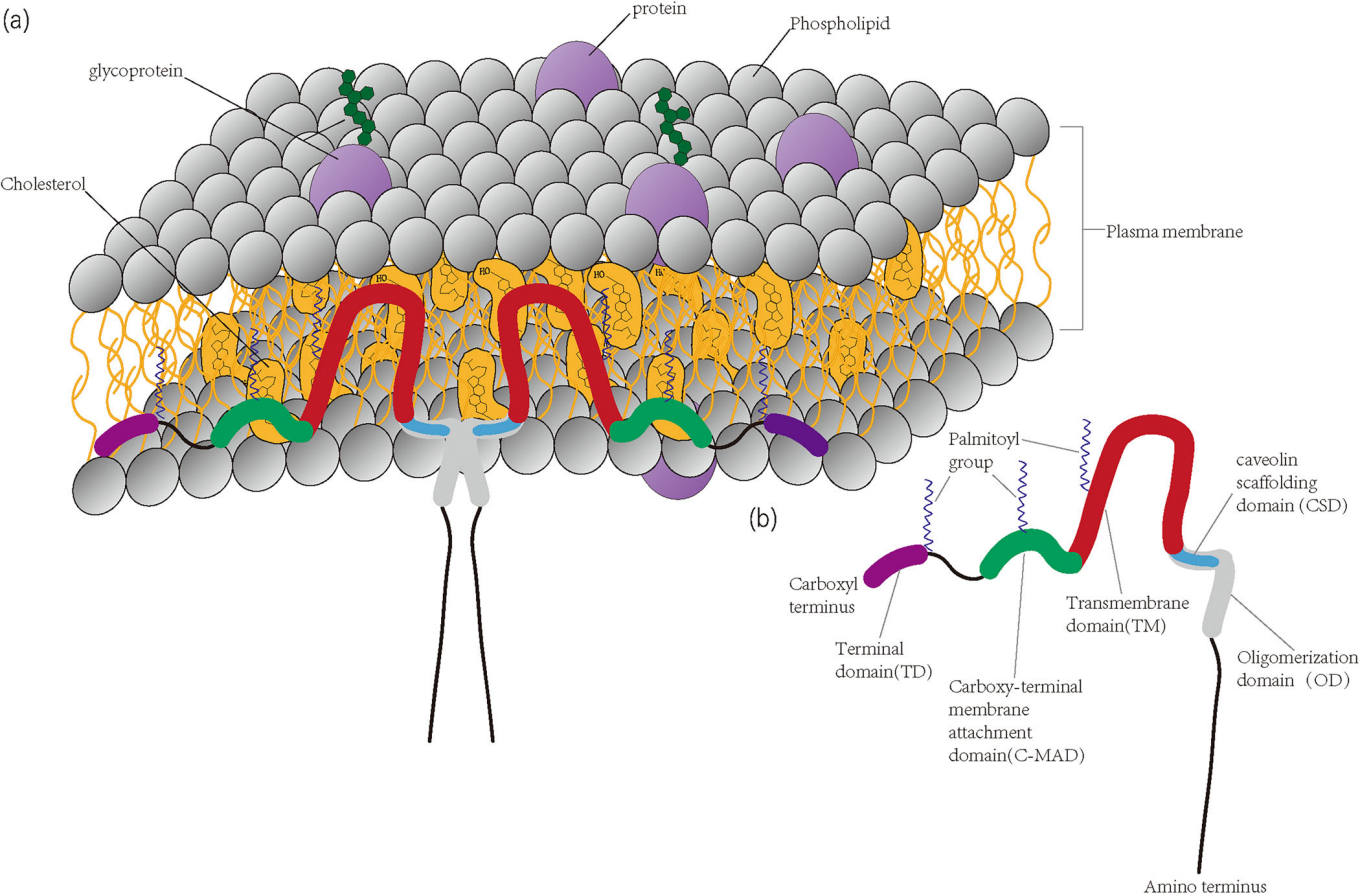
Primary structure and topology of Cav‐1. (a) The predicted membrane topology of Cav‐1. For simplicity, two Cav‐1 monomers are depicted forming a dimer; however, in reality, approximately 14‐16 monomers typically self‐associate to form a single Cav homo‐oligomer, which constitutes the caveolar assembly unit, similar to the clathrin triskelion. Both the amino‐terminal and carboxy‐terminal domains are oriented toward the cytosolic side of the plasma membrane, with a hairpin loop structure embedded within the membrane bilayer. Adapted from Williams et al. [4]. (b) The structural domains of Cav‐1. It is important to note that the CSD is also referred to as the amino‐terminal membrane‐attachment domain.

The Cav family includes three isoforms: Caveolin‐1 (Cav‐1, also known as VIP21), Caveolin‐2 (Cav‐2), and Caveolin‐3 (Cav‐3). Cav‐1 was initially discovered by Glenney et al. as the key protein characteristic of caveolae, with an approximate molecular weight of 22 kD [15] (The structural diagram of Cav‐1 is shown in Figure 1). This protein is widely distributed across various cell types, and can be found in structures such as the endoplasmic reticulum, vesicles, Golgi apparatus, and various other cytoplasmic sites. Cav‐1 is especially prevalent in fully differentiated cells, including endothelial cells, adipocytes, smooth muscle cells, and various types of epithelial cells. Cav‐1 is also expressed in several immune cells [16], including human and rat polymorphonuclear neutrophils (PMNs), murine macrophages, mast cells, as well as lymphocytes from bovine and murine species, and human dendritic cells and T‐cell lines. Cav‐2, which often co‐localizes and is co‐expressed with Cav‐1, is present in cells such as endothelial cells, adipocytes, and fibroblasts. For accurate membrane targeting, Cav‐2 depends on Cav‐1, and the Cav‐2 gene is situated in close proximity to Cav‐1, within the same chromosomal region (7q31.1 in humans) [17]. While Cav‐2 and Cav‐1 share certain similarities, Cav‐3 is more closely related to Cav‐1 in terms of protein sequence. Cav‐3 is a muscle‐specific protein, predominantly expressed in myocytes, including smooth muscle cells, skeletal muscle cells, and cardiac muscle cells.

## 3 Cav‐1 Function

### 3.1 Involvement of Cav‐1 in Immune Regulation and Inflammation

Cav‐1 is pivotal in controlling host immune responses and defending against infections, primarily through the regulation of immune cells and inflammatory pathways, particularly through interactions with lipopolysaccharides (LPSs) and pathogen recognition receptors. While Cav‐1 does not directly recognize antigens, it influences the cellular response to antigenic stimuli, triggering immune reactions by interacting with bacterial pathogens and their endotoxins, and facilitating the entry of pathogens into host cells. Cav‐1 expression is involved in immune cell reactions to LPS, contributing to the host's ability to resist infections [13]. Research by Medina et al. [18] demonstrated that B‐lymphocytes from Cav‐1‐deficient mice had a diminished secretion of immunoglobulin G3 in response to LPS induction. This deficiency also impacted the expression of Cav‐1 on PMNs, affecting PMNs activation and LPS‐induced lung injury. Cav‐1 has also been shown to regulate LPS‐induced cytokine production in macrophages and is involved in leukocyte migration as well as the tissue damage associated with inflammation [19, 20]. Toll‐like receptors (TLRs), particularly TLR‐4, recognize LPS from Gram‐negative bacteria and are involved in the regulation of the immune system's response to pathogens. Cav‐1 influences LPS‐induced TLR‐4 expression across various cell types. Overexpression of Cav‐1 enhances TLR‐4 expression in response to LPS, and both proteins are co‐localized within caveolae, where they interact to trigger inflammatory responses [21].

Cav‐1 has distinct roles in different immune cells, and its absence can impair immune and inflammatory responses [22]. It affects monocyte differentiation into macrophages by regulating β2‐integrin, Janus kinase signaling, and transcriptional activation pathways [23, 24, 25]. Moreover, the knockdown or knockout of Cav‐1 inhibits the activation of extracellular signal‐regulated kinase (ERK), which impacts nuclear translocation and reduces the transcriptional activity of early growth response‐1. This disruption affects the macrophage colony‐stimulating factor signaling pathway, thereby influencing macrophage differentiation [26, 27]. Additionally, Cav‐1 prevents apoptosis in macrophages induced by endoplasmic reticulum stress by activating the p38 mitogen‐activated protein kinase (MAPK) pathway [28]. During B‐lymphocyte activation and antigen presentation, Cav‐1 plays a regulatory role in B‐cell immune responses by interacting with Src, glycosylphosphatidylinositol, and other junctional proteins, influencing antigen transport, antigen presentation, and B‐cell receptor‐mediated signaling [18]. In T lymphocytes, Cav‐1 is involved in co‐stimulatory signaling and immune synapse activity, promoting T cell proliferation and cytokine secretion by mediating interactions between membrane‐associated guanylate kinase protein 1 (CARMA1) and cluster of differentiation 26 (CD26), also known as dipeptidyl peptidase‐4. Additionally, Cav‐1 facilitates the polarization of lipid rafts between T cells and antigen‐presenting cells (APCs) and supports actin polymerization [29]. Furthermore, Cav‐1 plays a significant role in mediating PMNs activation, adhesion, transendothelial migration, and activation, all of which are crucial for regulating inflammatory responses [16].

### 3.2 Involvement of Cav‐1 in Endocytosis

Caveolae can act as endocytotic structures, akin to lattice protein‐encapsulated porelets, which concentrate and facilitate the translocation of specific proteins. However, cells can uptake proteins through various endocytosis pathways, with caveolae‐mediated endocytosis being just one of them [30]. Endothelial cells utilize caveolae for efficient transmembrane transport, and it seems that some pathogens have developed strategies to hijack caveolae as a means of entering eukaryotic cells [31]. Furthermore, the internalization of transforming growth factor‐β (TGF‐β) receptors is linked to caveolae. Studies have demonstrated that while receptor internalization via lattice proteins is associated with signal transduction, internalization through caveolae is connected to receptor recycling. Therefore, the pathway used for receptor uptake influences whether the receptor undergoes signaling or is targeted for degradation [32].

### 3.3 Involvement of Cav‐1 in Cholesterol Homeostasis

Enriched in cholesterol, caveolae contain Cav‐1, one of the few proteins that binds to cholesterol with both specificity and strength [33]. The proper formation of caveolae relies on free cholesterol [34], which also plays a role in regulating the Cav‐1 promoter [35]. Cav‐1 also influences intracellular cholesterol homeostasis; dominant‐negative Cav‐1 mutants lead to the accumulation of intracellular free cholesterol and a decrease in both cholesterol synthesis and efflux from the cell [36]. Furthermore, caveolae play a key role in reverse cholesterol transport, facilitating the transfer of excess free cholesterol to the plasma via the uptake of high‐density lipoprotein particles. In addition, caveolae are involved in the absorption of cholesteryl esters from the plasma [37, 38].

### 3.4 Involvement of Cav‐1 in Signal Transduction

Lipid raft signaling is critical in the development of various diseases, such as cardiovascular diseases, prion diseases, human immunodeficiency virus, Parkinson's, Alzheimer's, and systemic lupus erythematosus (SLE) [39]. Caveolae, a specific type of lipid raft enriched with Cav proteins (Cav‐1, ‐2, and ‐3), form a distinct signaling platform that plays a central role in signaling events during disease progression. Consequently, Cav has become a significant target for therapeutic strategies aimed at treating and preventing these conditions.

Caveolae function as a signaling hub by clustering and organizing various signaling molecules, a concept referred to as the “caveolae signaling hypothesis” [40]. Several types of signaling molecules, including endothelial nitric oxide synthase (eNOS), G protein subunits and receptor, small G proteins, and non‐receptor tyrosine kinases [41], interact with Cav‐1 through its CSD peptides. Cav‐1 has the ability to suppress the activation and downstream pathways of various proteins such as H‐Ras, c‐Src, MAPK, and eNOS [42, 43, 44, 45]. While the CSD of Cav‐2 differs from that of Cav‐1, making its role as a potential signaling modulator unclear, the CSD of Cav‐3 closely resembles Cav‐1's CSD. The caveolae formed by Cav‐3 segregate and regulate various signaling molecules, such as adrenergic receptors, eNOS, G proteins, protein kinase C (PKC) isoforms, components of the myotonic dystrophy‐glycoprotein complex, and Src‐family kinases [43, 46, 47, 48, 49].

### 3.5 Involvement of Cav‐1 in Oxidative Stress (OS)

OS, resulting from a disruption in the balance between the production and elimination of reactive oxygen species (ROS), plays a critical role in cellular senescence by damaging macromolecular structures. OS has been linked to various degenerative diseases, such as osteoarthritis [50]. Research has demonstrated [51] that Cav‐1 is involved in OS‐induced senescence, with OS promoting an increase in Cav‐1 expression, while antioxidants have the opposite effect, reducing Cav‐1 levels. The expression of Cav‐1 may also vary depending on the duration of OS exposure, whether it is acute or chronic. Caveolae, a specialized membrane microdomain crucial for signal transduction, are considered to be a major site for ROS production. Due to its sensitivity to OS, Cav‐1 expression, as well as its posttranslational modifications, subcellular localization, transport, and functional activity, are all influenced by OS. Furthermore, studies focused on OS response have shown that exogenous oxidative damage, even at sublethal levels, can lead to an upregulation of Cav‐1 expression [52].

## 4 The Role of Cav‐1 in AIDS

### 4.1 Cav‐1 and Rheumatoid Arthritis (RA)

RA is a long‐term inflammatory condition driven by the immune system, marked by the gradual damage of cartilage and bone, accompanied by the infiltration of immune cells and the proliferation of synovial cells. Schönle et al. [53] demonstrated that Cav‐1‐deficient T cells preferentially differentiate into regulatory T cells (Tregs), and the absence or dysfunction of Tregs generally contributes to the development of AIDs. In the context of RA's inflammatory infiltration, CD26 on T cells serves a crucial co‐stimulatory function in the activation of CD4^+^ T cells when encountering memory antigens, thereby initiating antigen‐specific immune reactions [54]. Cav‐1 acts as a co‐stimulatory ligand for CD26, binding to CD26 to promote T cell proliferation in a manner dependent on the T cell receptor/CD3 signaling pathway [29]. Cav‐1, expressed on APCs, acts as a binding chaperone for CD26, and the presence of exogenous CD26 can activate downstream signaling pathways involving Cav‐1 [55, 56]. Therefore, modulating the interaction between activated T cells and APCs by targeting Cav‐1 might offer a novel therapeutic strategy for treating autoimmune and other immune‐mediated disorders.

Synovial fibroblasts (SFs) are central to the pathogenesis of RA and share malignant tumor cell‐like characteristics, including uncontrolled proliferation, resistance to apoptosis, and high invasiveness. MicroRNAs (miRNAs) regulate SFs' biological functions, offering potential for both diagnosis and therapeutic interventions in RA, with Cav‐1 potentially serving as a target for miRNA regulation. A study by Supin Li et al. [57] demonstrated that miR‐192 expression is reduced in the synovial tissues of individuals with RA. Reintroducing miR‐192 expression was shown to suppress the proliferation of SF and promote apoptosis by targeting Cav‐1. These findings suggest that the disruption of the miR‐192/Cav‐1 pathway may be a critical factor in the progression of RA, offering a potential novel therapeutic target for the condition.

A hallmark of RA is the ability of SFs to promote the chemoattractant‐driven influx of inflammatory cells into the joints [58]. This process is mediated by a chemokine gradient, and as such, inhibiting the multistep migration of inflammatory cells to the inflamed area could provide therapeutic benefit for RA. Cav‐1 influences inflammation in a context‐dependent manner, responding to cellular stress, growth factors, or hormonal signals, while regulating the balance between pro‐inflammatory and anti‐inflammatory cytokines [59, 60, 61]. This regulatory function of Cav‐1 helps control the progression of RA, providing a potential therapeutic avenue. In synovial fluid‐derived SFs, Cav‐1 mediates the expression of C–C chemokine ligand 2/monocyte chemoattractant protein‐1 [62], which then promotes monocyte migration into the joint cavity, differentiation into macrophages, and the development of RA synovitis through the production of pro‐inflammatory cytokines and other mediators [63]. Nonetheless, the exact function of Cav‐1 in the inflammatory processes associated with RA remains poorly understood and requires further exploration.

In chronic inflammatory conditions, Cav‐1 is essential in suppressing angiogenesis and facilitating endothelial cell apoptosis, mainly through its involvement in the formation of signaling complexes that include the double‐chain high molecular weight kininogen (HKa) receptor [64]. Methotrexate treatment for RA has been found to influence the re‐expression of genes associated with growth and apoptosis, including Cav‐2, suggesting that regulating the Cav‐2 gene could impact cell growth, proliferation, or apoptosis [65]. Furthermore, the cytokine interleukin‐1 (IL‐1) has emerged as a key therapeutic target in RA, with Cav‐1 playing a role in the vesicle‐mediated transport of the IL‐1 receptor [66].

In the clinical management of RA, long‐term or high‐dose GC therapy is linked to a range of notable adverse effects. A novel approach using dexamethasone‐carbon nanotube coupling aims to improve intracellular drug delivery by enhancing Cav‐1‐dependent endocytosis. This strategy allows for greater drug uptake and more efficient intracellular release at lower drug concentrations, potentially reducing side effects linked to prolonged GC use [67].

IκB kinase‐β (IKK‐β), a key kinase in the nuclear factor kappa‐B (NF‐κB) signaling pathway, is critical for phosphorylating NF‐κB and initiating the transcription of inflammatory mediators involved in inflammatory osteolysis [68, 69]. Targeting functional residues of IKK‐β is being explored as a therapeutic strategy for various diseases. Its impact on osteoclasts through Cav‐1 in the context of bone loss diseases like RA remains a critical area of research [70]. Studies with IKK‐β cysteine 46‐A transgenic (IKK‐β C46A) mice, in which cysteine 46 is mutated to alanine, have shown increased expression of Cav‐1 and membrane‐bound prostaglandin E2 synthase‐1 (mPGES‐1), promoting osteoclast differentiation and osteolysis, which exacerbates inflammatory bone destruction in RA [71].

### 4.2 Cav‐1 and SLE

SLE is an autoimmune disorder that targets multiple organs, distinguished by the dysregulated activation of various lymphocytes, the production of autoantibodies, and widespread organ involvement. Despite extensive research, the exact mechanisms behind its pathogenesis remain unclear.

Serum Cav proteins have emerged as potential biomarkers for various diseases. In SLE, the levels of Cav‐1 and Cav‐3 in the serum were found to be elevated when compared to healthy controls, whereas Cav‐2 was undetectable [72]. These findings suggest that Cav‐1 and Cav‐3 levels may hold diagnostic value for SLE. However, no substantial link has been found between their levels and disease activity in SLE patients, suggesting that additional research is needed to fully elucidate the clinical significance of Cav‐1 and Cav‐3 in this context [72].

The pathogenesis of SLE involves the activation of macrophages by activated lymphocyte‐derived DNA (ALD‐DNA), which triggers an inflammatory response [73, 74]. While ALD‐DNA on its own is typically internalized inefficiently, extracellular high mobility group protein B1 has been shown to enhance this process through a Cav‐1‐dependent receptor‐mediated endocytosis pathway. This mechanism results in the rapid accumulation of ALD‐DNA in vivo, offering insights into the pathogenic processes in SLE [75].

GC are frequently employed in the treatment of SLE, exerting their effects mainly through binding to membrane‐bound glucocorticoid receptors (mGCRs) or interactions with intracellular receptors. Recent studies have identified mGCR on the surface of human B‐lymphocytes and monocytes, with a notable increase in mGCR‐positive monocytes found in SLE patients. Although the binding of GC to mGCR on the cell membrane does not result in the upregulation of Cav‐1 or its co‐localization with mGCR in caveolae, Cav‐1 plays a crucial role in the translocation of these membrane receptors, highlighting its role in receptor‐mediated processes [76].

### 4.3 Cav‐1 and Sjögren Syndrome (SS)

SS is a long‐lasting autoimmune disorder that predominantly impacts the exocrine glands, resulting in symptoms like dry mouth and dry eyes, often accompanied by lymphocytic infiltration. While SS can also affect multiple organ systems, a reliable serological diagnostic marker is yet to be identified. Research on the role of Cav in SS is limited. However, it has been observed that the autoantigen tripartite motif‐containing protein 21 (also known as Ro52), which is present in SS patients, is recognized by anti‐Ro/SS‐related antigen A antibodies. Interestingly, studies have shown that Ro52 does not co‐localize with various cellular markers such as the proteasome subunit Rpt5, the caveolae component Cav‐1, endosomal markers like Ras‐related protein Rab5, Rab7, early endosome antigen 1, or the lysosomal marker lysosome‐associated membrane glycoprotein 2. These findings suggest that Ro52 does not reside within mitochondria, proteasome‐rich structures, minor fossa, endosomal compartments, or lysosomes, indicating that its exact structural organization requires further investigation [77].

### 4.4 Cav‐1 and Anti‐Neutrophil Cytoplasmic Antibody‐Associated Vasculitis (ANCA)

ANCA‐associated vasculitis (AAV) is a group of necrotizing vasculitides characterized by minimal or absent immune complex deposition. It mainly targets small to medium‐sized blood vessels and has the potential to affect various organ systems. Although advancements in immunosuppressive therapies have significantly improved life expectancy, the 5‐year mortality rate remains high, reaching 28% in some studies. Individuals with AAV face a higher likelihood of developing co‐morbid conditions, including infections, heart disease, cancer, and chronic kidney disease [78]. Cav‐1, an intracellular signaling pathway chaperone, has been linked to various fibrotic, vascular, and malignant diseases [41]. Research has shown that a genetic variant of Cav‐1, specifically the single‐nucleotide polymorphism rs4730751, is linked to elevated risks of all‐cause mortality, and higher risks of death from infections, cardiovascular disease, and accelerated cancer progression in AAV patients [79]. However, the potential of this genetic variant as a prognostic biomarker for AAV requires further investigation.

### 4.5 Cav‐1 and Systemic Sclerosis (SSc)

SSc, or scleroderma, is an autoimmune connective tissue disorder that primarily affects the skin and mucosal tissues, characterized by fibrosis and sclerosis. Key features of the disease include autoimmune alterations and fibrosis, primarily driven by the activation of fibroblasts, leading to the overproduction of extracellular matrix components and collagen in multiple tissues. The TGF‐β pathway is critical in driving fibroblast activation and is central to the development of fibrotic diseases [80, 81]. Cav‐1, a protein involved in cellular signaling, is crucial for regulating TGF‐β receptor internalization [32, 82] and has emerged as an important target for antifibrotic therapies.

In SSc, reduced expression of Cav‐1 enhances TGF‐β signaling, which subsequently further suppresses Cav‐1 levels at both the mRNA and protein levels, creating a feedback loop that amplifies fibrotic signaling and accelerates the progression of tissue fibrosis. Additionally, Cav‐1 expression is significantly reduced in the lung and skin tissues of SSc patients [83]. This suggests that restoring Cav‐1 function could be a promising therapeutic strategy for SSc and other fibrotic diseases. The lowered expression of Cav‐1 in the skin tissue of SSc patients has prompted researchers to adopt Cav‐1‐deficient mice as an innovative preclinical model for investigating scleroderma [84]. Furthermore, TGF‐β downregulates Cav‐1 expression and stimulates increased levels of pigment epithelium‐derived factor (PEDF) in dermal fibroblasts, which inhibits angiogenesis in SSc [85]. Racial differences have been observed in SSc susceptibility and severity, with black patients exhibiting higher TGF‐β expression and lower Cav‐1 levels compared to white individuals, highlighting the need for personalized treatment strategies [86].

Beyond TGF‐β, Cav‐1 is involved in other pathways that regulate the pathological changes in fibrotic diseases. A study by Rebecca Lee et al. [87] demonstrated that monocytes from SSc patients lacked Cav‐1, which disrupts the regulation of chemokine receptors (CCR1, CCR2, and CCR3) through mitogen‐activated extracellular signal‐regulated kinase (MEK)/ERK and Src/Lyn signaling pathways. This leads to enhanced migration and fibrotic differentiation of these monocytes, indicating that targeting Cav‐1 may represent a novel strategy to modulate signaling and chemokine receptor expression in fibrotic diseases. Cav‐1 has also been recognized as a susceptibility gene that regulates tissue fibrosis in SSc [88].

Although dermal fibrosis is a defining characteristic of SSc, the depletion of subcutaneous adipose tissue is another striking clinical feature [89]. In SSc patients, the loss of Cav‐1 and peroxisome proliferator‐activated receptor gamma (PPARγ) from adipocytes impedes lipogenic differentiation, resulting in the reduction of subcutaneous fat. However, CSD has been shown to reverse this process, suggesting that Cav‐1 and PPARγ collaborate to regulate the balance between fibrosis and adipogenesis. The absence of both Cav‐1 and PPARγ in SSc patients disrupts this equilibrium [90].

Microvascular alterations are frequently observed in SSc and play a significant role in the development of symptoms like Raynaud's phenomenon, finger ulcers, pulmonary hypertension, and capillary dilation [91]. Pericytes, a type of vascular cell, acquire a contractile phenotype under SSc conditions and are involved in microvascular dysfunction and fibrosis. Cav‐1 is abundant in pericyte membranes, where it contributes to the regulation of vascular tone and pulmonary fibrosis. Cav‐1 deficiency in pericytes impairs nitric oxide and calcium signaling, contributing to endothelial dysfunction, disrupted myogenic tone, and abnormal endothelial proliferation, leading to thickened alveolar septa in SSc patients [92]. Thus, Cav‐1‐deficient pericytes may contribute to microangiopathy and fibrosis in SSc. However, CSD's ability to serve as an alternative to Cav‐1 in reversing microangiopathy and fibrosis resulting from low Cav‐1 levels provides a protective effect [93, 94, 95]. Reese et al. [96] have demonstrated that CSD and its subregions inhibit microvascular leakage by directly regulating tyrosine kinases and affecting NO/ROS signaling, and may indirectly regulate fibrosis through effects on endothelial cells.

Cav‐1 also serves as a negative regulator of pro‐proliferative and oncogenic proteins, including those involved in breast cancer, a common malignancy in SSc patients. Cav‐1 regulates TGF‐β/Smad signaling pathways, which are essential for the development of tissue fibrosis and the progression of cancer. By modulating Cav‐1's CSD, it is possible to inhibit TGF‐β‐induced Smad phosphorylation, which could potentially reduce breast cancer risk in SSc patients [97]. Future research into Cav‐1‐mediated TGF‐β/Smad signaling could offer valuable insights into the connection between SSc and breast cancer, offering potential strategies for managing these diseases.

### 4.6 Cav‐1 and Connective Tissue Disease‐Associated Interstitial Lung Disease (CTD‐ILD)

CTD‐ILD is among the most prevalent and severe clinical complications associated with CTDs. It is frequently seen in patients with RA, SSc, SS, dermatomyositis/polymyositis, and other conditions. ILD can arise at any stage of CTD progression and presents with a variety of clinical manifestations, including respiratory symptoms like shortness of breath and cough, which gradually worsen and significantly impair the quality of life of affected individuals. Pulmonary arterial hypertension (PAH) is a significant complication of ILD. Cav‐1 plays a vital role in safeguarding pulmonary artery endothelial cells (PAEC) from excessive proliferation, abnormal migration, resistance to apoptosis, and inflammation. Exogenous IFN reduces Cav‐1 expression, activates signal transducers and activators of transcription 1 (STAT1) and protein kinase B (AKT), and disrupts the cytoskeletal structure of PAEC. These effects are linked to mechanisms associated with autoimmunity and autoinflammatory conditions contributing to PAH [98].

Cav‐1 has been closely linked to lung diseases, including the development of lung mesenchymal fibrosis, where its expression is downregulated. In RA‐ILD, the reduction in Cav‐1 levels serves as an indicator for whether primary alveolar type I epithelial cells undergo a transition to a mesenchymal phenotype. This epithelial‐to‐mesenchymal transition contributes to the fibrosis of alveolar epithelial cells post‐injury, providing novel perspectives on the mechanisms underlying ILD and informing future research on the factors influencing ILD models [99]. In animal models of RA‐ILD, Cav‐1 and TGF‐β1 serve as critical biomarkers, with Cav‐1 expression being significantly decreased and TGF‐β1 levels increased [100]. These proteins help regulate the relevant signaling pathways, which could represent potential therapeutic targets for treating RA‐ILD.

In SSc, ILD stands as a primary contributor to both morbidity and mortality, with Cav‐1 playing a crucial role in modulating monocyte function and signaling. Low expression of Cav‐1 in monocytes accelerates the development of ILD by influencing the migration and phenotype of these cells [101]. This results in excessive aggregation of fibroblasts, which promotes fibrosis. The increased expression of α‐smooth muscle actin (α‐SMA) during pulmonary fibrosis further enhances collagen production. Cav‐1 regulates collagen expression in cultured lung fibroblasts both in vitro and in vivo, and during pulmonary fibrosis [94, 102]. In SSc‐ILD, diminished Cav‐1 expression in fibroblasts triggers the activation of signaling molecules that promote enhanced collagen production and the overexpression of α‐SMA. CSD peptides have been shown to inhibit the production of tenascin‐C and collagen in both normal and fibrotic lung fibroblasts from SSc patients. Additionally, CSD peptides prevent epithelial cell apoptosis, reduce inflammatory cell infiltration, and protect against tissue morphological alterations, while also inhibiting the activation of signaling molecules involved in lung fibrosis. These findings suggest that CSD peptides may offer promising therapeutic potential for pulmonary fibrosis [103].

Adipose‐derived mesenchymal stem cells (MSCs) in SSc are known to be pro‐fibrotic and inhibit adipogenesis. Treatment with CSD peptides inhibits these functions, indicating that Cav‐1 is crucial in regulating MSC differentiation in the context of fibrotic diseases [104]. In SSc‐ILD patients, Cav‐1 expression is diminished in leukocytes, particularly in PMNs and monocytes/macrophages, resulting in altered signaling pathways that drive the advancement of pulmonary fibrosis [95]. Increased levels of TGF‐β and TNF‐α in both blood and tissues of SSc patients contribute to the reduced expression of Cav‐1 in monocytes, whereas treatment with CSD peptides restores Cav‐1 levels in these cells, suggesting that CSD peptides may help alleviate SSc‐associated ILD [105]. Patients with SSc and lower Cav‐1 expression in monocytes are at an elevated risk of developing ILD and experience more severe lung manifestations. This could explain why African American patients have a higher susceptibility to developing SSc‐ILD compared to Caucasians, highlighting Cav‐1's role in promoting pulmonary fibrosis by regulating signaling pathways, cell migration, and fibroblast differentiation [86, 106].

In normal lung fibroblasts, the MEK/ERK signaling pathway controls collagen expression [107]. Reduced Cav‐1 levels promote enhanced activation of the MEK/ERK pathway, leading to excessive collagen production—an important characteristic in the progression of SSc‐ILD. Increased levels of PKC‐ε activate MEK/ERK and promote collagen synthesis, whereas PKC‐α induces Cav‐1 expression, which in turn suppresses MEK/ERK activation and diminishes collagen production. These opposing effects in signaling pathways offer new perspectives for understanding pulmonary fibrosis in SSc [94].

Clinical studies comparing Cav‐1 concentrations in sputum samples from SSc patients and healthy controls have shown that Cav‐1 is significantly reduced in SSc patients, with even lower amounts found in those with SSc‐ILD. However, no clear associations have been observed between sputum Cav‐1 levels and disease activity, duration, or subtype. Further research may reveal whether sputum Cav‐1 levels can serve as a reliable biomarker for diagnosing SSc‐ILD [108].

### 4.7 Cav‐1 and Autoimmune Disorders of the Nervous System

Multiple sclerosis (MS) is a complex AID of the central nervous system (CNS), marked by blood–brain barrier (BBB) disruption and extensive infiltration of activated immune cells. The experimental autoimmune encephalomyelitis (EAE) model, a widely accepted surrogate for studying MS, is commonly utilized in research [109]. Cav‐1 has distinct roles in various neurological disorders, potentially offering new therapeutic strategies for inflammatory neurological diseases. For instance, in cerebral ischemia‐reperfusion injury, Cav‐1 plays a protective role by preserving the integrity of the BBB and inhibiting the breakdown of tight junctions (TJs) [110]. In contrast, Cav‐1 negatively regulates neurogenesis following a stroke, with its downregulation facilitating neuronal differentiation in ischemic stroke, potentially improving clinical outcomes [111].

A key aspect of MS pathology is the movement of encephalitogenic leukocytes from the bloodstream into the CNS tissue. Cav‐1 deficiency notably reduces the migration of these T cells into the CNS parenchyma while still maintaining immune activation in peripheral lymphoid organs [112]. As a result, Cav‐1 deficiency offers protective effects against MS, diminishing both clinical symptoms and neuroinflammation. Conversely, elevated Cav‐1 levels exacerbate MS development. The Wnt/β‐catenin signaling pathway plays a vital role in preserving the integrity of the BBB in healthy individuals. Inhibition of this pathway in CNS endothelial cells can result in increased expression of Cav‐1 and vascular cell adhesion molecule‐1, thereby enhancing endothelial cell transcytosis and exacerbating the symptoms of MS [113].

Meanwhile, the presence of Cav‐1 in brain endothelial cells contributed to the aggregation of the neuroinflammatory chemotactic CXCL10 into cytoplasmic reservoirs, which induced the transcellular migration of CD4^+^ T cells, further exacerbating inflammation [114]. Immune‐mediated demyelination in the cerebral cortex causes damage to microvessels, resulting in the breakdown of TJ proteins within affected microvessels. Cav‐1 regulates the migration of these TJ proteins, with both a reduction and increase in Cav‐1 affecting BBB integrity. TJ proteins including occludin and claudin‐5 are internalized by Cav‐1‐dependent endocytosis. In particular, the endocytosis of occludin involves a specific coiled‐coil structure within Cav‐1's C‐terminal domain [115]. When TJs are compromised, lymphocytes can cross the vascular barrier, initiating tissue inflammation. Interestingly, the remodeling of these junctions occurs early in MS but does not depend on caveolae at that stage. Additionally, in MS models lacking caveolae, the infiltration of Th1 lymphocytes into the CNS is reduced, indicating that Cav‐1 is necessary for Th1 cell migration across the BBB during autoimmune neuroinflammation [116].

Additionally, Cav‐1 interacts with CD147 in a range of physiological and pathological events associated with MS [117]. Cav‐1 serves as a key structural component of myelin, with increased gene expression during myelin formation in oligodendrocytes and Schwann cells. A loss of Cav‐1 expression has been associated with neurodegeneration, suggesting that the purinergic complex upstream of the Cav‐1 gene may serve as a binding site for inflammatory transcription factors, representing a novel susceptibility locus in MS pathophysiology [118, 119].

Elevated immunoreactivity for Cav‐1 and Cav‐2 was observed in ventricular tubular cells, specific astrocyte subpopulations, and inflammatory cells within the spinal cord [120]. Notably, Cav‐3 is only elevated in reactive astrocytes, indicating that the presence of these Cavs may be involved in regulating signaling pathways in the affected cells [120]. Cav‐1 has been linked to disease severity and incidence in murine models of EAE, suggesting its involvement in CNS‐directed lymphocyte trafficking and its potential as a therapeutic target for neuroinflammatory diseases [112, 116].

Research has indicated that the increase in sphingosine‐1‐phosphate receptor 2 in specific areas of the CNS, particularly in female mice, disrupts the formation of adhesion junctions via Cav‐1‐mediated endocytosis. This breakdown leads to altered BBB permeability, which may contribute to disease progression and could help explain the higher incidence of MS in females [121].

Experimental autoimmune neuritis (EAN), another model of T‐cell‐mediated AIDs such as Guillain–Barré syndrome, involves peripheral nerve demyelination and inflammatory cell infiltration. In the EAN model, Cav‐1 expression is significantly upregulated in the sciatic nerve, where it plays a role in molecular transport, nitric oxide production, and activation of eNOS. Cav‐1 also influences the apoptosis of inflammatory cells in the sciatic nerve [122]. Furthermore, the phosphorylation of Cav‐1 (p‐Cav‐1) has been observed in the sciatic nerve and its perineurium during inflammatory cell infiltration. This phosphorylation activates intracellular signaling pathways, resulting in cell death and the clearance of infiltrating inflammatory cells, a feature common to many AIDs [123]. The p‐Cav‐1 plays a critical role in apoptosis within the lesions observed in EAN, offering valuable insights into its involvement in inflammatory cell death and highlighting its potential as a therapeutic target for AIDs.

### 4.8 Cav‐1 and Autoimmune Uveitis (AU)

AU is a common inflammatory condition of the eye that may manifest either as a component of a systemic autoimmune disease affecting multiple organs or as an isolated uveitic disorder without systemic involvement. The pathogenesis of AU is closely linked to autoimmune processes, primarily driven by T‐lymphocytes, with CD4^+^ T cells, particularly Th17 and Th1 cells, being central to the progression of the disease. Th17 cells are especially implicated in disease recurrence.

Recent studies have suggested a link between Cav‐1 and various ocular diseases, such as autoimmune uveitis, primary open‐angle glaucoma, and diabetic retinopathy. However, the exact function of Cav‐1 in preserving normal vision remains largely uncharted [124]. A key pathological feature of autoimmune uveitis is the disruption of the blood–retinal barrier (BRB), which occurs when T‐cells, typically targeting self‐antigens, invade the retina—an immune‐privileged site. This disruption of the BRB is believed to result from changes in the cellular and tissue surface structures. Increased Cav‐1 expression has been observed at the outer limiting membrane, a critical component of the outer BRB. This elevated Cav‐1 expression is believed to contribute to BRB disruption, potentially through signaling pathways like integrin signaling, the TNF receptor pathway, and the upregulation of focal adhesions [125].

### 4.9 Cav‐1 and Autoimmune Thyroid Disease (AITD)

AITD, which includes Hashimoto's thyroiditis (HT) and Graves' disease (GD), stands as the most common organ‐specific autoimmune disorder. It is marked by lymphocytic infiltration of the thyroid gland and an increase in thyroid‐specific autoantibodies in the bloodstream. A key component in thyroid function, Cav‐1 is part of a complex known as the thyroxisome, which includes thyroid peroxidase, Dual oxidase, and Cav‐1. This complex resides at the apical membrane of thyroid cells, where it plays a crucial role in anchoring the hormone‐synthesizing components to the apical membrane. Cav‐1 participates in thyroid hormone synthesis and in regulating OS, which protects cells from damage and apoptosis [126]. HT and GD result from Th1 and Th2 immune responses, respectively. However, Cav‐1 expression in AITD varies depending on the type of immune response (Th1/Th2). Th1 cytokines, including interferon‐gamma (IFN‐γ) and IL‐1α, downregulate Cav‐1 expression, while the Th2 cytokine IL‐4 does not produce the same effect [127].

HT, a Th1‐dominant autoimmune thyroid disorder, presents with lower Cav‐1 expression, which correlates with elevated levels of IFN‐γ and IL‐1β. This is associated with a decrease in the levels of the autophagy marker protein LC3B‐II. Additionally, Knockdown of Cav‐1 in thyroid follicular cells results in decreased LC3B‐II expression, suggesting that the absence of Cav‐1 inhibits autophagic activity in these cells when exposed to Th1 cytokines. This may represent a novel pathogenic mechanism in HT [128].

GD is associated with Graves' ophthalmopathy (GO) in about one‐third of individuals affected by the condition. GO, also referred to as thyroid‐related ophthalmopathy or hyperthyroid synophthalmia, is an autoimmune disorder characterized by orbital adipocytosis and inflammation. The precise pathogenesis of GO remains unclear, though OS is considered a significant contributing factor. Cav‐1 is essential in modulating OS within adipocytes in GO. As an important protein in controlling the adipocyte calcium channel, Cav‐1 is essential for biological processes like glucose transport and fat synthesis. In GO adipocytes, OS leads to the downregulation of Cav‐1, which is accompanied by increased expression of hypoxia‐inducible factor‐1α (HIF‐1α) and Deiodinase 3. This disruption affects glucose transport via Glut‐4 and exacerbates OS through the NADPH oxidase 2 and eNOS pathways, highlighting Cav‐1's complex regulatory role in GO. Therefore, Cav‐1, a vital regulator of adipocyte homeostasis, offers potential as a promising therapeutic target for managing GO [129].

### 4.10 Cav‐1 and Autoimmune Myocarditis (AM)

AM is a prevalent cardiovascular condition often triggered by viral infections, characterized by either limited or diffuse inflammatory lesions in the myocardium. This condition primarily affects children and young adults. The disease progression can vary significantly; while most patients respond well to treatment, some individuals with severe cases may develop myocardial fibrosis, which can lead to dilated cardiomyopathy, ultimately resulting in impaired cardiac function or even heart failure.

In rats, experimental autoimmune myocarditis (EAM) provides an important model for investigating autoimmune heart disease and closely mirrors human giant cell myocarditis. Research has shown that Cav‐1 overexpression is linked to macrophage apoptosis and increases the susceptibility of fibroblasts and epithelial cells to apoptotic triggers. Additionally, low levels of Cav‐2 and Cav‐3 are initially found in the ventricular myocardium, but their expression increases markedly as the animals age. This observation led to the hypothesis that Cav‐1, in particular, plays a significant role in myocarditis, especially in macrophage‐mediated inflammatory responses within the heart. Ahn et al. [130] provided new insights by demonstrating that the expression of both Cav‐1 and Cav‐2 was notably increased in heart lesions within the EAM model, while Cav‐3 levels remained unchanged. This differential expression suggests that Cav‐1's role in stimulating second signaling molecules in macrophages and some cardiomyocytes may contribute to cell proliferation or death, providing a deeper understanding of the pathological mechanisms in autoimmune myocarditis.

## 5 Conclusions

**Figure 2:**
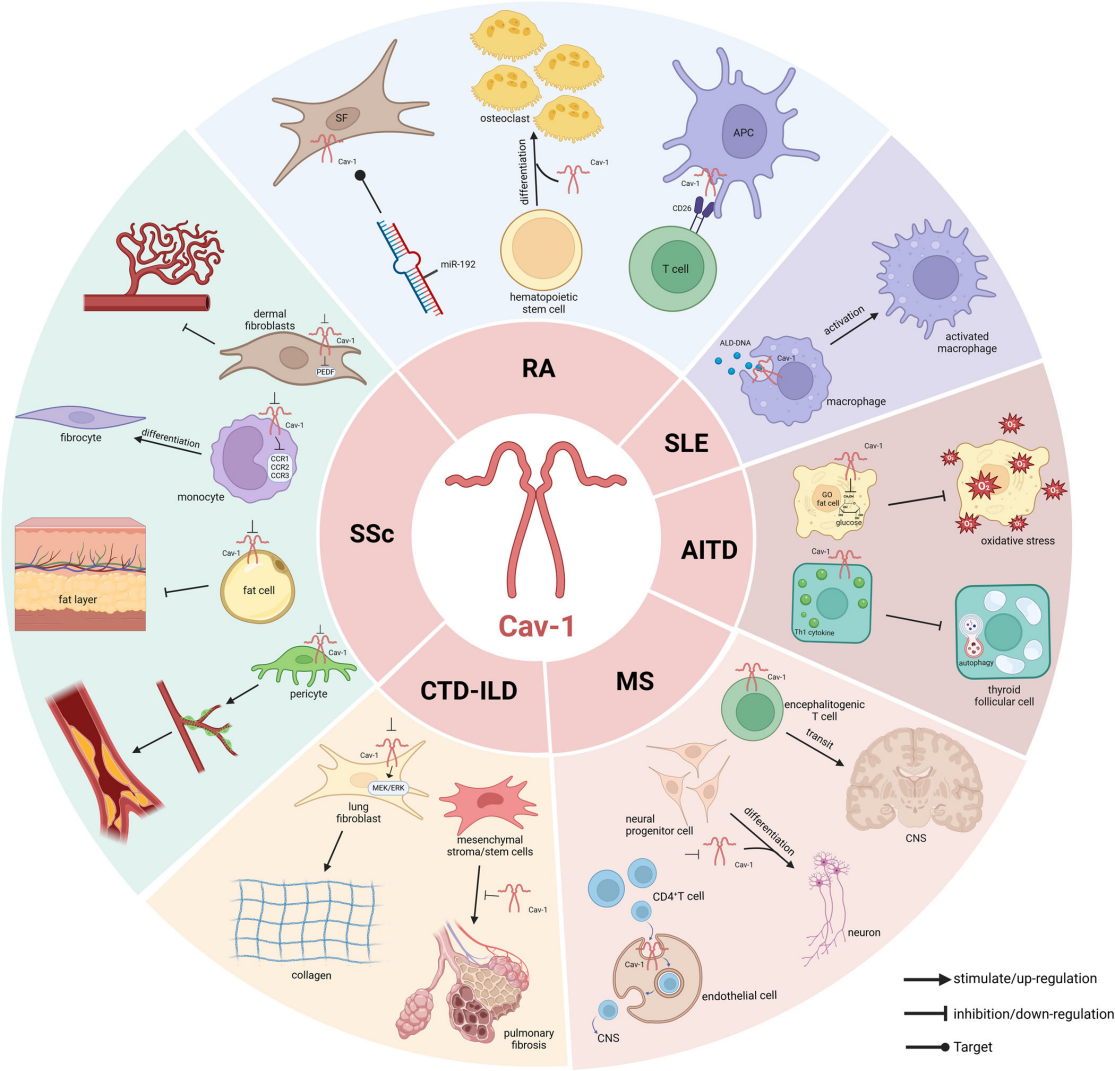
Mechanism of action of Cav‐1 in AIDs across related cells (created with BioRender. com). (Clockwise order) (1) In RA, the role of Cav‐1 is highlighted in SFs, osteoclasts, T cells, and APCs. (2) In SLE, the function of Cav‐1 in macrophages is summarized. (3) In AITD, Cav‐1's role in GO fat cells and thyroid follicular cells is outlined. (4) In MS, the involvement of Cav‐1 in encephalitogenic T cells, neurons, and CNS endothelial cells is summarized. (5) In CTD‐ILD, Cav‐1's role in mesenchymal stroma/stem cells and lung fibroblasts is described. (6) In SSc, the function of Cav‐1 in pericytes, fat cells, monocytes, and dermal fibroblasts is briefly outlined.

Cav‐1 is a crucial cell membrane protein that participates in various cellular functions, including signal transduction, material transport, and the maintenance of cell structure. Cav‐1 exhibits critical roles in various AIDs, as a major protein that constitutes inflammatory and immune cells, and can serve as a mediator of multiple signals and exert effects in rheumatic diseases by regulating the translocation of related signaling molecules and influencing multiple signals and pathways (Table 1 and Figure 2). Given its potential role in AIDs, Cav‐1 may serve as a clinical biomarker for diagnosis and monitoring. However, this area of research requires further clinical studies to validate its practical application. In addition, targeted drugs may be the key to treating the diseases in the future.

**Table 1:** Mechanisms of action of Cav‐1 in AIDs.

Autoimmune diseases	Cell/animal models	Machine	Reference
Rheumatoid arthritis	T cell	Cav‐1‐deficient T cells differentiate preferentially into Tregs	[53]
		Cav‐1 promotes T cell activation by functioning as a costimulatory molecule	[29, 55]
	SFs	miR‐192 targeting Cav‐1 affects proliferation and apoptosis in SFs	[57]
	Osteoclast	Increased Cav‐1 promotes osteoclast differentiation and osteolysis	[70, 71]
Systemic lupus erythematosus	Macrophage	Cav‐1‐dependent receptor‐mediated endocytosis pathway leads to quick and massive accumulation of ALD‐DNA in vivo and activation of macrophages to induce inflammatory responses	[75]
Systemic sclerosis	Dermal fibroblast	Downregulation of Cav‐1 expression induces increased PEDF expression in dermal fibroblasts and inhibits SSc angiogenesis	[85]
	Monocyte	Lack of regulation of Cav‐1 in monocytes results in upregulation of expression and function of chemokine receptors, enhancing migration toward fibrotic differentiation	[87]
	Fat cell	Loss of Cav‐1 from adipocytes and reversal of otherwise inhibited lipogenic differentiation by Cav‐1 CSD results in a reduction of the subcutaneous fat layer	[90]
	Pericyte	Pericyte deficiency of Cav‐1 promotes microvascular dysfunction and fibrosis	[92]
Connective tissue disease‐associated interstitial lung disease	PAEC	Reduced Cav‐1 expression in PAEC by IFN contributes to PAH through STAT1, AKT activation, and cytoskeletal disruption	[98]
	RA‐ILD animal model	Cav‐1 is a protein biomarker for RA‐ILD, and Cav‐1 expression was significantly reduced	[100]
	Lung fibroblast	Cav‐1 regulates collagen expression in lung fibroblasts and lung fibrosis, with low Cav‐1 levels leading to increased MEK/ERK activation and higher collagen production	[94, 102]
	Mesenchymal stroma/stem cells	Cav‐1 inhibits the pro‐fibrotic and anti‐adipogenic effects in adipose‐derived mesenchymal stromal/stem cells from SSc	[104]
Multiple sclerosis	Neurons	Downregulation of Cav‐1 promotes neuronal differentiation and alleviates clinical symptoms in ischemic stroke	[111]
	Encephalitogenic T cells	Cav‐1 deficiency significantly attenuates encephalitogenic T cells transit to the CNS parenchyma and has a significant protective effect against MS	[112]
	CNS endothelial cells	Increased Cav‐1 expression promotes endothelial cell transcytosis and exacerbates the clinical manifestations of MS	[113, 114]
	EAE	Cav‐1 acts as an active regulator of CNS‐directed lymphocyte trafficking	[112, 114, 116]
	EAN	Cav‐1 expression is significantly elevated in the EAN sciatic nerve	[122]
Autoimmune thyroid disease	Thyroid follicular cells	Lack of Cav‐1 expression inhibits autophagic activity in thyroid follicular cells exposed to Th1 cytokines	[128]
	GO fat cells	Cav‐1 regulates glucose supply in GO fat cells and reduces OS	[129]
Autoimmune myocarditis	Macrophages、cardiomyocytes	Increased Cav‐1 expression stimulates second signaling molecules in macrophages and some cardiomyocytes, leading to cell proliferation or cell death.	[130]

Abbreviations: AKT, protein kinase B; ALD‐DNA, activated lymphocyte‐derived DNA; CNS, central nervous system; CSD, Caveolin scaffolding domain; EAN, experimental autoimmune neuritis; ERK, extracellular signal‐regulated kinase; GO, graves' ophthalmopathy; HIF, hypoxia‐inducible factor; MEK, mitogen‐activated extracellular signal‐regulated kinase; NF‐κB, nuclear factor kappa‐B; OS, oxidative stress; PAEC, pulmonary artery endothelial cell; PEDF, pigment epithelium‐derived factor; PG, prostaglandin; PI3K, phosphatidylinositol‐3‐kinase; PK, protein kinase; SFs, synovial fibroblasts; STAT1, signal transducers and activators of transcription 1; Tregs, regulatory T cells.

Cav‐1 is mainly involved in various immune cell types, such as T cells, B cells, and macrophages, where it modulates cell signaling and immune reactions, influencing immune cell activation, proliferation, and apoptosis. In AIDs, Cav‐1 not only regulates classical receptor signaling pathways but also impacts inflammatory responses through interactions with newly identified cytokine receptors, suggesting new avenues for Cav‐1‐targeted therapies. Furthermore, Cav‐1's role in intercellular communication, including its regulation of immune responses through membrane microdomains, vesicular trafficking, and cytokine receptor interactions, deepens the understanding of its function in AIDs. Cav‐1 emerges as a promising therapeutic target for AIDs, with potential in regulating immune tolerance and reducing inflammation. Future research should focus on its interactions with other immune molecules and cytokine receptors to further clarify its role in AIDs and refine Cav‐1‐based treatment strategies.

Ultimately, Cav‐1's involvement in AIDs highlights its potential as a therapeutic target, and continued research may lead to breakthroughs in treatment, advancing novel therapeutic approaches. Therapeutic strategies to target Cav‐1 for the treatment of AIDs may include: (1) Cav‐1 inhibitors: therapeutic agents that work by blocking the expression or function of Cav‐1, thereby influencing intracellular signal transduction pathways. For example, Cav‐1 promotes endothelial cell cytophagy, and its inhibitors help attenuate T‐cell transit to the CNS, which may be useful for the treatment of MS. (2) Cav‐1 agonists: Enhancing Cav‐1 function may be beneficial in some cases, such as in SSc, where Cav‐1 activation promotes angiogenesis and inhibits fibrosis, potentially slowing the progression of the disease. (3) Gene editing: Using gene editing techniques such as CRISPR/Cas9, the Cav‐1 gene can be precisely modified to modulate Cav‐1 function at the molecular level. (4) Drug delivery systems: Developing systems that can specifically deliver drugs to Cav‐1‐expressing cells, such as exosome‐based drug delivery systems, could enhance therapeutic efficacy while minimizing side effects.

Notably, the therapeutic potential of Cav‐1 must be carefully evaluated, as we observed conflicting findings regarding its role in pro‐neuroinflammation versus neuroprotection. Cav‐1 deficiency significantly reduces T‐cell migration to the CNS parenchyma [112]. However, in cerebral ischemia‐reperfusion injury, Cav‐1 exerts a protective effect by maintaining the integrity of the BBB and inhibiting the disruption of TJs [110]. Additionally, while inhibition of Cav‐1 alleviates MS, it accelerates cancer progression [6, 8], underscoring the need for targeted strategies that account for cell type, disease stage, and the presence of other comorbidities in patients.

The study of Cav‐1 and the occurrence and development of AIDs is still in the primary stage. With the deepening of the study, Cav‐1 could open new avenues for diagnosing and treating AIDs, enhancing drug sensitivity, and emerging as a novel target for the treatment of various AIDs.

## Author Contributions

**Yanan Gao:** conceptualization (supporting), data curation (lead), investigation (equal), project administration (lead), visualization (lead), writing – original draft (lead), writing – review and editing (equal). **Liangyu Mi:** conceptualization (supporting), methodology (lead), supervision (lead), writing – original draft (supporting), writing – review and editing (equal). **Yingren Deng:** investigation (equal), writing – review and editing (equal). **Zhaojun Jia:** investigation (equal), writing – review and editing (equal). **Miaomiao Zhao:** investigation (equal), writing – review and editing (equal). **Yuting Hu:** investigation (equal). **Yuli Ji,** investigation (equal). **Xiaoyao He:** investigation (equal). **Ke Xu:** Conceptualization (lead), supervision (supporting). All authors provided feedback on earlier versions and gave their final approval for the manuscript.

## Ethics Statement

The authors have nothing to report.

## Consent

The authors have nothing to report.

## Conflicts of Interest

The authors declare no conflicts of interest.

## Data Availability

Data sharing is not applicable to this article as no new data were created or analyzed in this study. No data was used for the research described in the article.
